# Retracing the path of evolution: polymorphisms of *aspA* codon 363 shape the fitness of *Yersinia pestis*

**DOI:** 10.1080/22221751.2025.2532700

**Published:** 2025-07-10

**Authors:** Kai Song, Ruichen Lv, Leiming Shen, Yarong Wu, Xiuwei Qian, Yiming Cui, Yumeng Wei, Yanbing Li, Yanting Zhao, Wenwu Yao, Yafang Tan, Yanping Han, Yujing Bi, Zongmin Du, Florent Sebbane, Yujun Cui, Ruifu Yang, Yajun Song

**Affiliations:** a State Key Laboratory of Pathogen and Biosecurity, Academy of Military Medical Sciences, Beijing, People’s Republic of China; b Huadong Research Institute for Medicine and Biotechniques, Nanjing, People’s Republic of China; c College of Life Sciences, Fujian Agriculture and Forestry University, Fuzhou, People’s Republic of China; d College of Pharmaceutical Sciences, Soochow University, Suzhou, People’s Republic of China; e Department of Laboratory Medicine, Xiangya Hospital of Central South University, Changsha, People’s Republic of China; f Inner Mongolia Agricultural University, Hohhot, People’s Republic of China; g Zhejiang Provincial Center for Disease Control and Prevention. Hangzhou, People’s Republic of China; h Univ. of Lille, CNRS, Inserm, CHU Lille, Institut Pasteur de Lille, U1019—UMR 9017—CIIL —Center for Infection and Immunity of Lille, Lille, France

**Keywords:** *Yersinia pestis*, *Aspa*, polymorphism, competition, fitness, evolution

## Abstract

*Yersinia pestis*, the etiologic agent of plague, is a genetically monomorphic pathogen. By screening 1085 published *Y. pestis* genomes, we identified an unusual mutation hotspot within codon 363 of the aspartate ammonia-lyase gene *aspA* with at least six alleles in the population. Our investigation delves into the significance of the polymorphism at the *aspA* codon 363 and its impact on the fitness of *Y. pestis* strains. Notably, we found that the dominating TTG allele (L363), resulting in AspA inactivation, confers a fitness advantage to *Y. pestis* when competing with other bacteria inhabiting a similar niche, due to elevated expression of pesticin. However, the TTG strain exhibits fitness deficits under various stress conditions compared to the GTG strain (V363), which expresses an active AspA. Drawing from these observations, we propose an evolutionary hypothesis for *Y. pestis* at the *aspA* codon 363 locus. It is inferred that GTG represented the ancestral state at this locus. However, the TTG strain swiftly dominated populations in the early evolutionary stage of *Y. pestis*, likely owing to the V363L substitution that conferred a competitive advantage to *Y. pestis* over other bacteria. Subsequently, spontaneous mutations emerged, reinstating AspA activity, thereby offering fitness advantages across diverse environments and get fixed within the population. Our findings portraited a scenario of inactivating-restoring mutations relay in *Y. pestis* microevolution, shedding new lights on the evolutional dynamics of pathogens.

## Introduction

*Yersinia pestis*, the notorious agent behind plague, ranks among the most lethal and globally impactful pathogenic bacteria. Its presence spans diverse ecological niches encompassing humans, rodents, fleas, and various natural environments such as soil, facilitating its survival, propagation, and persistence throughout its lifecycle [1]. Notably, genomic comparative analyses trace the emergence of *Y. pestis* from the enteric pathogen *Yersinia pseudotuberculosis* to around 7000 years ago [2]. Key genetic acquisitions, notably the *pla* gene, especially the subsequent I259 T substitution in Pla, play pivotal roles in the pathogenicity and epidemic dissemination of *Y. pestis* [3]. Further enhancements to its flea-borne transmission potential involve the inactivation of *ureD*, *rcsA*, and *pde*2, paired with the acquisition of *ymt* [4–6].

The evolutionary landscape of *Y. pestis* reveals over thirty phylogroups within five primary branches [7]. Notably, the first pandemic stems from an extinct lineage labelled 0.ANT4 [8], while the second and third pandemics trace back to strains within branch 1, representing the most widely dispersed lineage within *Y. pestis*. Branches 1–4 radiated from the Big Bang node that emerged in the early fourteenth century, immediately preceding the Black Death [9]. Branch 0, a root lineage of *Y. pestis*, houses ancient strains identified so far and several “atypical” groups. Among these, the 0.PE7 group stands as the oldest among all modern *Y. pestis* natural isolates [7]. Remarkably, the 0.PE4 group, assigned to the biovar Microtus, exhibits high virulence in mice but reduced virulence in larger mammals like guinea pigs and humans [10].

Genomic analyses indicate that *Y. pestis* as a genetically monomorphic species, showing very limited genetic diversity, with a genome mutation rate estimated between 3.1 × 10^−9^–1.3 × 10^−7^ per site per year [7]. While the fixation of single nucleotide polymorphisms (SNPs) in the *Y. pestis* genome primarily occurs through neutral processes like genetic drift, our focus lies on codon 363 of YPO0348 (*aspA*), encoding aspartate ammonia-lyase (AspA, also known as aspartase) displaying significant polymorphism with six distinct alleles [7].

AspA, ubiquitous in procaryotes, plays a pivotal role in aspartate metabolism by catalyzing the deamination of aspartate to fumarate and ammonia, which can be integrated into gluconeogenesis and nitrogen assimilation, respectively [11]. AspA deficiency in bacteria hampers the utilization of exogenous aspartate and intracellular aspartate formed by transamination, disrupting its recycling into the tricarboxylic acid cycle [12]. Among the alleles of *Y. pestis* AspA, the codon at position 363 is highly polymorphic. Six different alleles have been reported so far: GTG (Val), TCG (Ser), TTT and TTC (Phe), ATG (Met), and TTG (Leu). Notably, the prevalent TTG codon in modern *Y. pestis* strains inactivates AspA enzymatic activity, contrasting with other alleles encoding active AspA variants [13,14]. This mutation possibly suggests a selective advantage, considering the rarity of aspartate deficiency in bacteria [15]. The presence of other alleles hints at reverted mutation events on *Y. pestis* AspA, indicating the potential role of natural selection in shaping *aspA* codon 363 diversity. Previous research has linked functional AspA to the avirulence of *Y. pestis* in humans, as observed in attenuated biovar Microtus strains where the encoding nucleotides at the locus represent an active AspA [14].

The diverse codons at *Y. pestis aspA* codon 363, corresponding to distinct AspA enzyme activities, likely establish connections between locus polymorphisms and *Y. pestis* fitness in varied environments. Therefore, we investigated this possibility in this study.

## Materials and methods

### Genome data collection

Genome assemblies and raw sequencing data (comprising both modern strains and ancient DNA) of *Y. pestis* were acquired from the NCBI GenBank and SRA databases as of July 2021. Low-quality reads (<Q20) were filtered using Trimmomatic (v0.38) [16], and the filtered sequencing data for modern strains were assembled with SPAdes (v3.13.0) to validate the reliability of public assemblies [17]. Our final dataset comprised 992 assemblies of modern strains and 93 ancient DNA sequences of *Y. pestis*. Besides, a total of 311 genome assemblies and 581 raw sequencing datasets of *Y. pseudotuberculosis* retrieved from the NCBI repository were also used.

### SNP identification and phylogenetic analysis

The assemblies were aligned to the CO92 chromosome reference (NC_003143.1) using MUMmer (v3.23) [18]. Core-genome SNPs were identified from the MUMmer alignments, excluding those in repetitive regions. A maximum-likelihood tree was generated from the identified SNPs using IQ-TREE (v1.6.5) [19] with the GTR + G model and ultrafast bootstrapping (bootstrap = 1,000). The resulting tree was visualized with the online tool iTOL (v6.8) [20]. Briefly, to ensure both clarity and representativeness in the phylogenetic reconstruction, we selected 992 modern *Y. pestis* genomes from a pool of over 3,000 strains included in our recent study [21]. For lineages containing only the TTG allele at codon 363 of *aspA*, representative genomes were randomly subsampled to minimize redundancy. Conversely, all lineages harbouring non-TTG alleles were retained in full to facilitate a comprehensive evaluation of allelic diversity across the phylogeny.

For modern *Y. pestis* strains, the allele state at codon 363 of *aspA* was determined from the SNP matrix and further validated using raw sequencing reads. Reads were aligned to the CO92 reference genome (GCF_000009065.1) using BWA-MEM (v0.7.17) [22] with default parameters, and variants were called using GATK HaplotypeCaller (v4.2.4.0) [23], requiring a minimum of ten supporting reads and an allele frequency > 0.9. For ancient DNA (aDNA), phylogroup assignment was based on previously published studies. We retrieved publicly available *Y. pestis* aDNA genomes published up to July 2021. Raw reads were processed using Trimmomatic v0.38 to remove reads shorter than 30 bp or with a Phred score below 20. The filtered sequencing data were mapped to the CO92 reference genome using BWA-MEM with default settings. Aligned reads were extracted using SAMtools v1.9 with the -bF 4 flag and subsequently merged. Reads with more than 10 soft- or hard-clipped bases were removed using samclip, and PCR duplicates were eliminated using Picard’s MarkDuplicates tool (v2.21.2). Mutation sites at the *aspA* 363 codon for each genome were subsequently identified with GATK's HaplotypeCaller module with a minimum threshold of three supporting reads and an allele frequency greater than 0.9. Positions that did not meet these criteria were manually inspected in the corresponding BAM files, with sites near read termini excluded to reduce the potential impact of terminal damage.

We also examined the allele state of codon 363 in the *aspA* gene across *Y. pseudotuberculosis*. A total of 892 strains were analysed, comprising 311 genome assemblies from NCBI GenBank and 581 raw sequencing datasets from the NCBI SRA database. Raw reads were trimmed using Trimmomatic and assembled with SPAdes (v3.13.0). All assemblies were aligned to the *Y. pestis* CO92 reference chromosome using MUMmer, and the codon 363 position in *aspA* was subsequently inspected to determine the corresponding allele as described above.

### Bacterial strains and growth conditions

The bacterial strains and plasmids used in this study are listed in Table S1. *Y. pestis* biovar Microtus strain 201, a human-avirulent strain while lethal in mice, was isolated from the rodent *Microtus brandti* in Inner Mongolia, China, and has a gene content almost identical to that of *Y. pestis* strain 91001 [10,24]. Strain 201 is a GTG strain belonging to phylogroup 0.PE4C and expresses an active AspA [13,14]. To generate the isogenic TTG mutant, allelic exchange was performed using a suicide plasmid pDS132, as illustrated in Figure S1. Briefly, a fragment of the *aspA* gene carrying the desired point mutation (e.g. TTG at codon 363) was PCR-amplified and cloned into pDS132 digested with SphI and SacI. The recombinant plasmid (pDS132_TTG) was electroporated into *E. coli* S17-λpir, which was then used as a donor strain for conjugation with *Y. pestis* strain 201. After overnight culture, the bacterial mixture was plated on Yersinia Selective Agar Base (YSAB) supplemented with 8 µg/ml chloramphenicol. Selected *Y. pestis* colonies were subcultured in LB broth and counter-selected on LB agar supplemented with 7% sucrose. Lastly, allelic exchange at the *aspA* locus was confirmed by Sanger sequencing. All strains were grown in LB or TMH liquid medium (Table S2), or on LB agar at 26°C (for *Y. pestis*) or 37°C (for *E. coli* or *Y. pseudotuberculosis*). The glycerol-preserved strains were inoculated into 5 ml of medium and fully activated overnight. The subcultured inocula in fresh medium were used in follow-up experiments. Chloramphenicol (34 μg/ml) was added to the media when needed.

### Aspa enzymatic activity detection

AspA activity was assessed using Nessler's reagent colorimetric method. Bacteria were grown to late-exponential phase, harvested, washed in PBS, then suspended to an optical density of OD_620_ = 2.5. Aliquots (1 ml) of bacterial suspensions were incubated with 50 mM L-aspartic acid or ddH_2_O (control) at 37°C. After 30 min of incubation, 0.4 ml Nessler’s reagent (0.09 M K_2_HgI_4_ and 2.5 M KOH) was added to each sample to detect ammonium production. A yellow–brown coloration indicated active AspA, whereas pale yellow reflected minimal or no activity. This assay enabled direct detection of AspA activity without protein purification.

### Growth curves

The *Y. pestis* strains were incubated in LB/TMH medium at 26℃ with shaking overnight for activation recovery. Inocula were subcultivated in fresh medium at 26℃. Samples with an optical density at 600 nm (OD_600_) of 1.0 were incubated in 150 ml Erlenmeyer flasks containing 60 ml medium of interest at a ratio of 1: 200. Triplicates were performed for each strain and OD_600_ of each sample was recorded at regular intervals during the growth cycle.

When the curve reached plateau, the data were collected for growth curve plotting. To avoid long and variable lag time, for all other specific-medium growth experiments, both strains were subcultured twice at 26°C in each specific medium prior to growth experiments to adapt to the conditions. The results of all growth curves were presented as the normalized areas under the growth curves, with the TTG strains as the standards. All experiments were performed three times independently with three replicates.

### Real-time cell analysis (RTCA) assay

The HeLa cells were cultivated in Dulbecco’s modified Eagle’s medium (DMEM, Gibco, USA) containing 10% fetal bovine serum at 37°C in a 5% CO_2_ incubator. 50 μl of the medium was pipetted into each well of the E-plate connected to the RTCA iCELLigence system to measure the baseline. 5 × 10^3^ HeLa cells were added into each well of the E-plate. The E-plate containing the cells was incubated for 30 min under room temperature, followed by being transferred to the RTCA iCELLigence system at 37°C in a 5% CO_2_ incubator. When cell proliferation came into the stable logarithmic growth period, *Y. pestis* cells were resuspended in the same medium and were added to the wells at a multiplicity of infection (MOI) of 10. The cell index was measured at 2 min intervals. The experiment was performed three times independently with three replicates.

### Competition assay

The GTG and TTG strains stored at −80℃ in 15% glycerol were incubated in liquid TMH/LB medium at 26℃ with shaking for 24 h for activation recovery. The cultures were subcultivated in fresh medium at 26℃ with shaking and once the OD_620_ reached 1.0, they were mixed in a certain proportion in each assay. The mixtures were incubated at a ratio of 1:20 in liquid media at 26℃ with shaking for 96 h subsequently or were transferred at the exponential phase for another passage every 8/12 h. In total, all the liquid cultures were maintained for up to 30/100 passages. In the meantime, a fraction of each passage (including the initial mixture as the starting population) was harvested by centrifugation and stored at −80℃ for genomic DNA extraction (QIAGEN DNA Extraction kit).

As for the competition assay *in vivo*, female mice were challenged by mixtures at a certain proportion via Subcutaneous injection (∼1000 CFU per mouse). Tissue samples (0.25 g) from spleens of mice (n = 10 for each group) were collected once the mice died and used for DNA extraction (QIAGEN tissue DNA Extraction kit).

Amplicon sequencing enables high resolution of a single nucleotide while maintaing high-throughtput capability for multiple competitive samples through deep sequencing. Thus, each genomic DNA was used as the template and high-fidelity PCR amplicon sequencing was performed using primers amplicon-F (5’ ACG ATA CTT GCA TTA CTA TG 3’) and amplicon-R (5’ GGT TAA GAT AAG TGA CGA TG 3’) with specific Barcodes. The frequency of the GTG and TTG strains in each passage or tissue sample was obtained by calculating the ratio of G: T at the target site of the amplicon.

To visually evaluate the fitness difference between the two strains, a competition index μ was introduced [25]:

$$\mu =\log10\;\left(\frac{\left(\mathrm{Tt}/\mathrm{Gt}\right)}{\left(T0/G0\right)}\right)$$

where T and G denote raw read counts from the amplicon of the TTG and the GTG strains, respectively. Terms (T_t_/G_t_) can be interpreted as the ratio of the absolute number of the TTG to the GTG strain at a time point (t), which is normalized to that at the initial time point (t_0_). Denary logarithm transform is used to determine the ratio μ. According to the meaning of the ratio μ, the points below the X axis imply that the GTG shows a competitive advantage, that is, the portion of the GTG strain can outnumber that of the TTG strain during the series passages.

### Viability of the two strains in macrophage

RAW cells were spread in 12-well plates at a density of 10^6^ cells per well and incubated overnight at 37℃ in an incubator containing 5% CO_2_ overnight before infection. The GTG and TTG strains were allowed to grow at 26℃ to the exponential growth phase (OD_620_ = 1.0, equivalent to a bacterial density of 2 × 10^8^ CFU/ml), followed by adding to the cells at a concentration at a multiplicity of infection (MOI) of 5. Plates were centrifuged at 700 × *g* for 5 min to facilitate bacterial contact with macrophages. Media were discarded and PBS was added to wash the cells after 30 min of infection. gentamicin 30 μg/ml was added to kill the bacterial cells which failed to enter the macrophages for 30 min and use the sample at the time as point 0 h. Cells were lysed in each well with 0.3% Triton X-100 for 15 min at 2, 4, 6, and 8 h, and the CFU count was performed on the two strains. The experiment was performed three times independently with three replicates.

### Mice challenge assay

Overnight cultures of the two strains were allowed to grow at 26℃ to the exponential growth phase (OD_620_ = 1.0), which were used to challenge mice (female BABL/c, 8 weeks old). The mice (*n* = 10) were challenged with the two strains subcutaneously or intravenously (∼ 1000 CFU per mouse) and mortality was recorded continuously for two weeks. Mice at the terminal stage of infection were euthanized by cervical dislocation following institutional guidelines and ethical regulations. Survival curves were analysed using the log-rank (Mantel–Cox) test. All the mice were handled as per the Guidelines for the Welfare and Ethics of Laboratory Animals of China. All experiments were performed twice independently.

### Survival assay under stressed conditions

Overnight cultures of the TTG and GTG strains were allowed to grow in TMH at 26℃ to the exponential growth phase (OD_620_ = 1.0), which were used for the survival assays of the two strains under certain stressed conditions. CFU of the culture above was counted as the control. Then, the cultures were transferred into the solutions simulating different stressed conditions such as acid (pH = 5.5, 30 min), cold shock (4℃), heat shock (50℃, 10 min), anaerobic, high salinity (0.5 M NaCl, 1 h), and oxidative stress (0.2 M H_2_O_2_, 10 min) for a period. CFU of each strain was counted at different time points. And the survival rate was the ratio of the CFU at a time point divided by that of control. All experiments were performed at least three times independently with three replicates.

### RNA extraction and data analysis

Total RNA was extracted using the PureLink™ RNA Mini Kit (Invitrogen, USA) for creating a cDNA library and transcriptomics sequencing. Differential expressions were expressed as the logarithm to base 2 of fold change (Log_2_FC). The genes with differential values more than twofold were applied to analyse the differential expression according to the *Y. pestis* 91001 genome annotation. Each sample included three biological replicates.

### Bactericidal assay

A bactericidal assay was established using *E. coli* cells expressing *Y. pestis* FyuA (DH5α-*fyuA*) from a pET22b vector to test if there was a difference between the two strains in killing *E. coli* cells expressing FyuA. Overnight cultures of the TTG and GTG strains and *Y. pseudotuberculosis* or DH5α-*fyuA* were allowed to grow to the exponential growth phase. Initial CFU was counted as the control. Mix *Y. pseudotuberculosis* or DH5α-*fyuA* with the TTG or GTG strain and the mixture was incubated at 26℃. The mixture was gradient-diluted and the diluted mixture was dripped onto the plate containing ampicillin (100 μg/ml). CFU of the *Y. pseudotuberculosis* or DH5α-*fyuA* was counted on the same plate for the comparison between the survival rate after the *E. coli* cells undergoing the bactericidal effect by the two strains. Meanwhile, the CFU of each strain was also counted on a yersinia selective agar base for the comparison between the survival rate after mixed with the *E. coli* cells. All experiments were performed at least three times independently with three replicates.

### Ethics statement

The animal study was conducted in accordance with institutional guidelines and ethical regulations of the Beijing Institute of Microbiology and Epidemiology (IACUC-IME-2023-001).

### Statistical analysis

All data of the experimental groups were obtained from three independent experiments and expressed as mean ± standard deviation. As appropriate, statistical analysis was applied using the Student t-test, Wilcoxon test, the one-way analysis of variance, or *chi*-square test. The one-way analysis of variance was performed when groups are more than two, the Student–Newman–Keuls-q test was applied for multiple comparisons, and chi-square test was used to analyse the comparison of the composition ratio between the two groups. Statistical significance was set as follows: *p < 0.05, **p < 0.01, ***p < 0.001, and ****p < 0.0001.

## Supplementary Material

Fig S1.jpg

Fig S5.jpg

Fig S6.jpg

Table S6.docx

Fig S2.jpg

Table S7.docx

Table S4.docx

Fig S4.jpg

Revised Table S3.xlsx

Table S1.docx

Table S2.docx

Table S5.xlsx

Fig S3.jpg

## Figures and Tables

**Figure 1.**
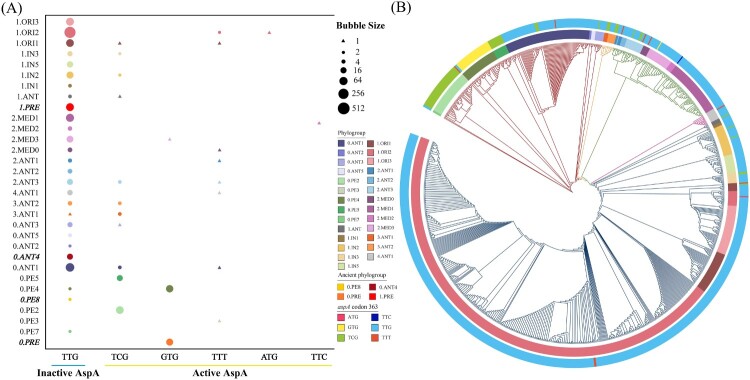
Polymorphism at codon 363 of *aspA* and phylogenetic relationships among modern *Y. pestis* strains. (A) Polymorphism at the *aspA* codon 363 in the form of a bubble plot. The size of the bubbles indicates the allele frequency for the genotypes, with log_2_-adjusted values (triangle denotes original frequency = 1). The ancient phylogroups are formatted in bold italic. (B) Maximum-Likelihood Tree of and the polymorphism at the *aspA* codon 363 modern *Y. pestis* strains. The inner ring is the Maximum-likelihood tree of 4293 single-nucleotide polymorphisms (SNPs) from 992 modern strains of *Y*. *pestis*. The outer ring is the polymorphisms at codon 363 in the *aspA* gene.

**Figure 2.**
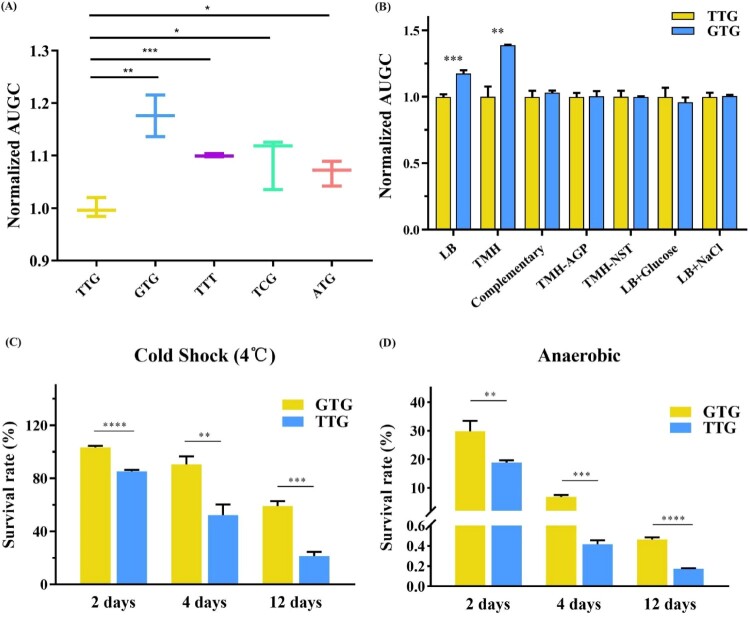
Differential phenotypes of the GTG and TTG strains. Normalized Area under the growth curves for all mutants in LB medium (A) and for GTG and TTG strains in various media (B) at 26°C. The composition of the defined TMH medium is described in Supplementary Table S2. TMH-AGP, TMH medium lacking D, Q, P, N, and E. TMH-NST, TMH medium lacking P, S, T, E, D, and NH_4_^+^. Complementary, growth curves of complemented strains in LB at 26°C. LB + Glucose, LB with 0.1 M glucose. LB-NaCl: LB with 0.5 M NaCl. (C) Survival rates of the two variants under cold shock. (D) Survival rates of the two variants under anaerobic condition. Statistical analysis was performed using t-test and one-way ANOVA. *P < 0.05; **P < 0.01; ***P < 0.001, ****P < 0.0001.

**Figure 3.**
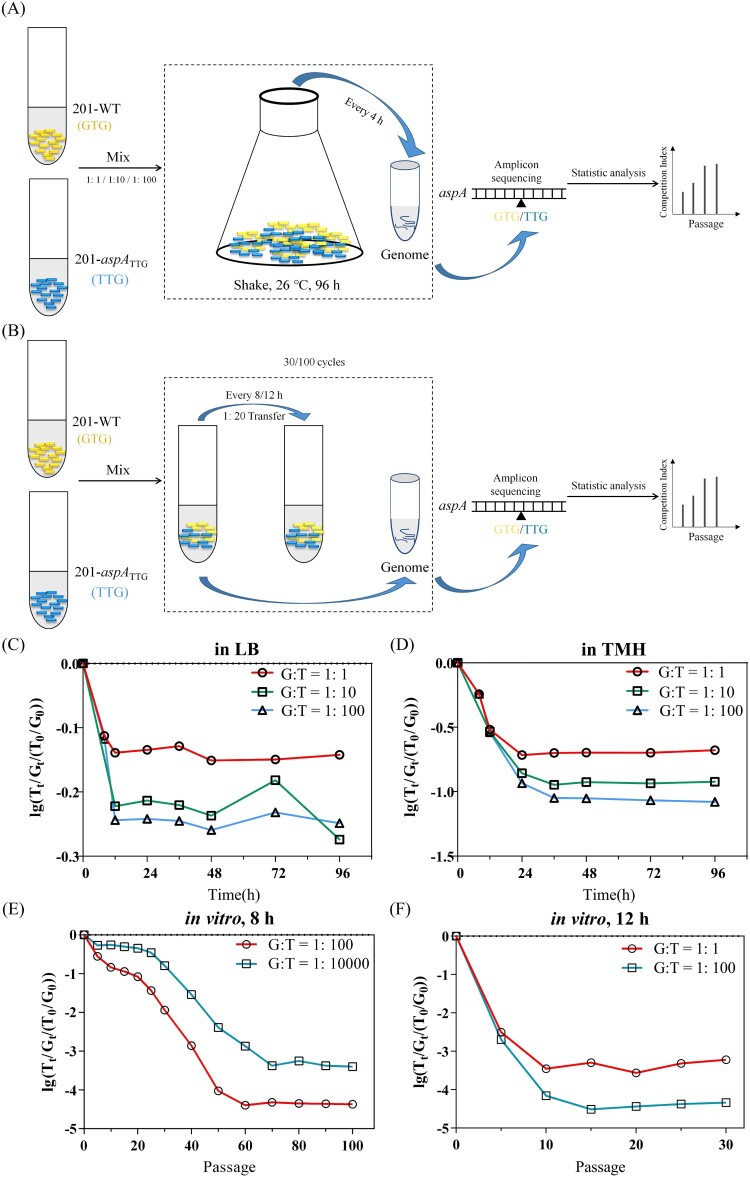
Competitive assay of co-culture of GTG and TTG variants *in vitro*. Schematic depiction experimental approach of competitive assay of the two strains with successive passaging (A) and continuous co-culture for 96 h with no passaging culture (B). Changes in the ratio μ of the two competitive strains successive co-cultured for 96 h in LB (C) or in TMH (D), or with continuous passaging culture every 8 h in LB (E), or every 12 h in TMH (F). The formula μ = lg (T_s_/W_s_ / (T_0_/W_0_)) indicates that points located under the horizontal axis show the fitness of the GTG variant is stronger than the TTG variant.

**Figure 4.**
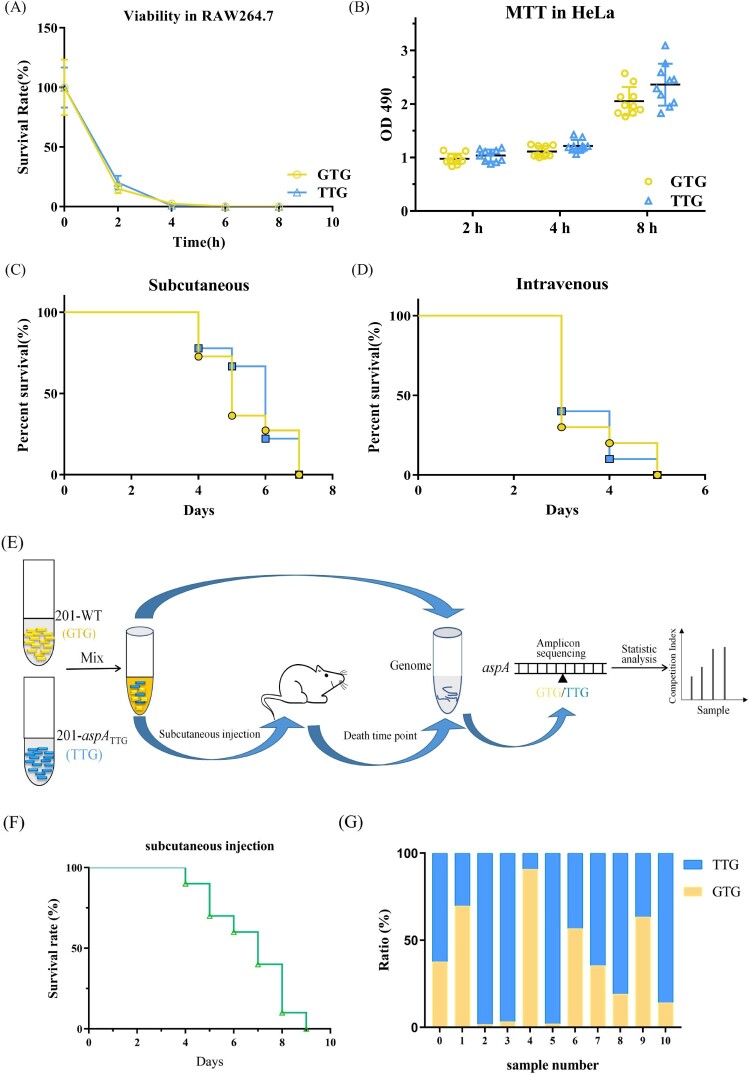
Virulence and *In vivo* competitive assay of the GTG and TTG variants. (A) Measure of the survival upon contact with RAW264.7 macrophages (*n* = 3). (B) Measure of the cytotoxicity after contact with HeLa cells using the MTT assay (*n* = 10). (C-D) Survival curve of mice challenged with GTG and TTG variants respectively through subcutaneous (C) and intravenous (D) injection (*n* = 10). Regardless of the experiment, the two strains did not display any difference (*P* > 0.05 in t-test and a Log-rank (Mantel-Cox) test). (E) Schematic depiction of the experimental approach for assessing the competitive fitness of the GTG and TTG strains *in vivo* in mice. (F) Survival curve of the mice challenged by the mixed culture of the GTG and TTG strains at an initial ratio of 1:1. (G) Results of *in vivo* competition between the GTG and TTG strains at an initial ratio of 1:1. The horizontal axis represents the sample numbers of different mouse individuals, where “0” represents the initial mixing ratio of the two strains. The vertical axis represents the ratio of the two strains in each mouse.

**Figure 5.**
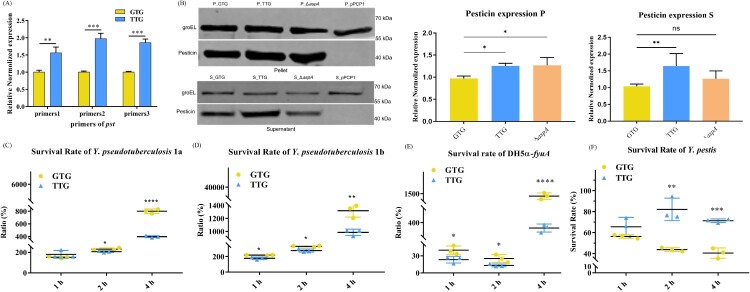
The higher expression level of pesticin correlates with a stronger bactericidal ability of the TTG variant. (A) qPCR confirmation of the expression of *pst*. (B) Western blotting confirmation of the expression of pesticin. The bar graphs show the statistical analysis of three replications of the WB results. Ratio of the *Y. pseudotuberculosis* strain 1a (C) and 1b (D) after incubated with the two variants. (E) Survival rate of DH5ɑ-*fyuA* after co-incubation with the two variants. (F) Survival rates of the two variants after co-incubation with DH5ɑ-*fyuA*. Statistical analysis was carried out using a t-test analysis of variance. *P < 0.05; **P < 0.01; ***P < 0.001; ****P < 0.0001.

**Figure 6.**
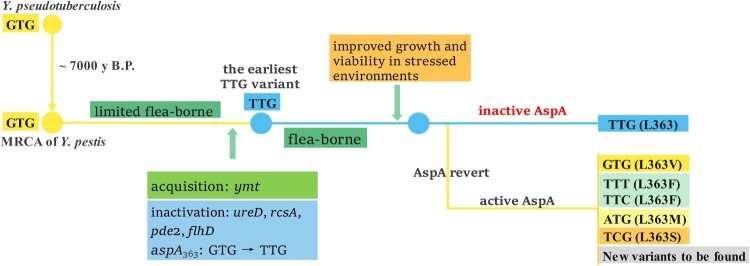
Hypothesis of *Y. pestis* evolutionary journey at *aspA* codon 363.

## Data Availability

The transcriptomic data reported in this paper have been deposited in the National Microbiology Data Center (NMDC) database (https://nmdc.cn/) under BioProject NMDC10018137 (https://nmdc.cn/submit/sra/overview/SUB1654477096622).

## Results

### Polymorphism at *Y. pestis* aspA codon 363

To decode the rationale behind the polymorphism observed at the *aspA* codon 363 locus in *Y. pestis*, we conducted a comparative analysis of *aspA* sequences from 992 modern and 93 ancient strains available in public databases (Table S3). Sequence alignment unveiled six alleles at codon 363 (GTG, ATG, TCG, TTT, TTC, and TTG), with TTG being the most prevalent (Figure 1(A), Table S4). Essentially, this locus presents five amino acids (Val, Met, Ser, Phe, and Leu) at the same AspA position. Simultaneously, a maximum-likelihood phylogenetic tree based on contemporary strain genomes was constructed (Figure 1(B)). The phylogenetic tree indicated multiple independent mutations at codon 363 during the evolutionary trajectory of *Y. pestis*.

Specifically, all the most ancient strains (0.PRE) and most within the oldest 0.PE lineages (0.PE2, 0.PE3, 0.PE4, 0.PE5) harbour an allele that preserving AspA activity. Notably, GTG at codon 363 is predominantly found in 0.PE4 lineage (Microtus). Sequence alignments reveal the conservation of GTG at codon 363 in all *Y. pseudotuberculosis* strains (Table S5), the immediate progenitor of *Y. pestis*, suggesting GTG as the ancestral state at the *aspA* codon 363 locus in *Y. pestis*. Conversely, the vast majority of more recent strains, including those from 0.ANT and lineages within branches 1-4, exhibit the TTG allele, known to disrupt AspA activity. Intriguingly, the TTG allele occupies the oldest modern lineage 0.PE7, suggesting a possible replacement of the ancestral GTG by TTG at this site. These data suggest that active AspA (GTG allele) characterizes the most ancient *Y. pestis* lineage [26]. However, it is rarely active in the most recent strains (TTG allele), while alternative alleles conducive to active AspA emerge in a fluctuating pattern.

### Active aspA provides *Y. pestis* with a growth advantage *in vitro*

In exploring the impact of *aspA* evolution in *Y. pestis*, we generated a Microtus strain 201 (0.PE4) replacing codon 363 (GTG) with TTG, TCG, ATG, TTT, or ATA (not included in the six alleles) as illustrated in Figure S1. Only the TTG mutant displayed no AspA activity (Figure S2), consistent with previous findings [13,14]. The TTG mutant with inactive AspA exhibited a significant growth deficit compared to the parental strain and the other mutants with active AspA when cultured at 26°C in LB medium (Figure 2(A) and Figure S3). This confirms the growth advantage provided by active AspA in *Y. pestis*. Furthermore, the growths of the TTG and GTG strain in different media were performed to determine whether there was a defect in growth of the TTG strain due to the loss of the AspA activity (Figure 2(B)). The difference in growth disappeared when the TTG strains harboured a parental AspA in LB media (Figure S4A and B). We further found that the mutant with an inactive AspA (i.e. a TTG codon 363) also has a reduced growth rate when cultured in the synthetic TMH medium [27], unless this later lacks sources of aspartate or other nitrogen sources [11] (Figure S4 C-E). Notably, under culture conditions containing 0.1 M glucose or 0.5 M NaCl (known to inhibit AspA activity [28,29]), the GTG strain exhibited growth kinetics indistinguishable from the TTG strain (Figure S4F and G). Altogether, the data suggested that AspA is important for *in vitro* growth of the *Y. pestis* Microtus strain.

### Active aspA is vital for the survival of *Y. pestis* under some environmental stresses

Given that *Y. pestis* encounters environmental stresses during its life cycle, including within the flea or possibly in soil, we tested whether AspA activity contributes to stress resistance. We notably compared the ability of the wild-type GTG strain (representing functional AspA alleles) and the TTG strain (serving as the inactive variant) to survive in a cold condition or in an anaerobic environment. There was a difference between the two strains after incubation under 4°C or anaerobic conditions, with the wild-type strain surviving significantly better than the mutant strain (Figure 2(C and D)). Overall, our results indicate that the active AspA confers a fitness advantage to *Y. pestis* under some stressful conditions, such as low temperature and anaerobic environments. This advantage may play a role in the survival of *Y. pestis* under natural pressures and contribute to the polymorphism of the *aspA* codon 363 through AspA-reverted mutations.

### Competitive advantage of GTG strain

Given that the GTG strain exhibited a growth advantage over the TTG strain when cultured in isolation, we conducted growth competition experiments in LB or THM media at 26°C. In these experiments, we utilized mixed inoculants containing GTG and TTG strains at 1:1, 1:10, or 1:100 (Figure 3(A)). In this scenario, the GTG strain showed a minor competitive advantage over the TTG strain, especially in TMH (Figure 3(C and D)). The curves depicting the μ-value plateaued after 24 h, implying that the ratio of the two strains in the mixed cultures has stabilized. This was because both strains reached a stationary period of growth. When the mixed cultures were continuously passaged in fresh media every 8 h in LB or 12 h in TMH (Figure 3(B)), the data also showed a clear and consistent dominance of GTG strain over TTG strain across all tested media and ratios, even under highly unfavourable conditions for the GTG strain (Figure 3(E and F)). Therefore, competition experiments *in vitro* demonstrated that the GTG strain is more adaptive in competition when co-cultured with the TTG strain, possibly due to its growth advantage over the TTG strain.

### GTG strain and TTG strain have similar virulence in mice and cells

Considering that an active AspA provides a growth advantage in different media, one may suggest that AspA activity may be important during an infectious process. This is why we compared the capacity of the Microtus WT strain (V363) and its isogenic mutant lacking AspA activity due to the V363L substitution to survive upon contact with RAW264.7 macrophage (Figure 4(A)), because *Y. pestis* may hide within the macrophages during the early stage of infection. We also compared the ability of the strains to be cytotoxic against Hela cells and to cause fatal plague in mice inoculated by subcutaneous or intravenous route. Regardless of the experiments performed, no difference was observed between the WT and mutant strain (Figure 4(B–D)), indicating that an active AspA is not essential for the extreme virulence of the *Y. pestis* Microtus strain in mice.

Therefore, we further tested whether AspA could provide a fitness advantage *in vivo* using a competitive assay in mice. To this end, groups of mice were inoculated with a mixture of a bacterial suspension composed of the wild-type GTG strain and the TTG mutant at an initial ratio of 1:1, and the ratio of each bacterial strain was determined in animals that were sacrificed at the terminal stage of infection (Figure 4(E–G)). The result shows that the mutant was not outcompeted by the wild-type *in vivo*, indicating that the activity of AspA does not provide any fitness in the mice model of plague.

### The v363l substitution confers a competitive advantage to *Y. pestis* when cultured with other bacteria by increasing pesticin expression.

Despite having a deficit AspA and fitness costs under certain circumstances, The TTG allele at the *aspA* codon 363 (L363 mutation) predominates in the most modern strains of *Y. pestis*, suggesting that the microevolution of the ancestral allele GTG into the allele TTG may provide a significant selective advantage. However, we found that the V363L substitution has multiple adverse effects. These unexpected results suggest that the selective advantage provided by the V363L substitution is not trivial. We performed a comparative transcriptomic analysis to identify a genetic signature to get a clue on the putative advantage provided by the V363L substitution. The analysis unveiled 60 genes that were up/down-regulated more than 2-fold between the wild-type and the mutant strain (Table S6). Among them, the *pst* encoding the pesticin was up-regulated significantly, which was confirmed by RT-qPCR (Figure 5(A)) and western blotting (Figure 5(B)). The pesticin is a bacteriocin against bacteria that produce the pesticin receptor (FyuA) but not the pesticin immunity protein (Pim). Therefore, one may suggest that the evolution of the V363L substitution has been selected to confer a colonization advantage in an environment colonized by bacteria that express FyuA but not Pim. To test this hypothesis, we performed a bactericidal assay by incubating *Y. pestis* Microtus displaying the TTG or GTG strain in the presence of *Y. pseudotuberculosis* 1a and 1b (which naturally produce FyuA but not Pim), or *Escherichia coli* expressing FyuA of *Y. pestis* [30]. In agreement with our hypothesis, we found that the TTG mutant strain (i.e. with inactive AspA) was a better killer of *E. coli* expressing FyuA and of *Y. pseudotuberculosis* than its parental strain (i.e. with active AspA) (Figure 5(C–E), Figure S5). Additionally, when co-incubated with DH5ɑ-*fyuA*, the TTG mutant strain also exhibited a higher survival rate than the wild-type GTG strain (Figure 5(F)). Altogether, the data suggest that the microevolution of the *Y. pestis* ancestral *aspA* allele GTG to TTG, leading to the inactivation of AspA activity due to the V363L substitution and thus overexpression of the pesticin, had been selected to colonize a niche occupied by other bacteria more efficiently. This may partially explain why the strains with the TTG allele, despite having a deficit AspA, still predominate in *Y. pestis* populations in nature.

## Discussion

Deciphering the selective pressures driving mutation hotspots and repeatedly drive the fixation of certain mutations remains a complex challenge, as does elucidating their biological implications in terms of biological function and evolution [31]. Under variable selective pressures, mutations causing phenotypic changes may be fixed by conferring different fitness advantages onto bacteria in varying environments [32]. We postulated intrinsic connections between specific loci polymorphisms and bacterial fitness. In fact, long periods of telluric survival have been confirmed in *Y. pestis* [1]. Therefore, the soil has been proposed as a reservoir for the persistence of *Y. pestis* in nature and a source of plague as well [33]. Outside of mammal hosts, it is crucial for *Y. pestis* to respond to diverse environmental stresses, such as low temperatures, limited nutrients, and niches in its vector fleas [1].

A single nucleotide substitution altering a protein can significantly influence the evolution of a species, particularly in bacteria [34]. Previous research has demonstrated that at least four amino acids occur in the codon 363 of *Y. pestis* AspA [14]. To further explore the polymorphism of the locus, we aligned the *aspA* sequences from published genome data of 992 modern strains and 93 ancient strains of *Y. pestis* [21], and 892 *Y. pseudotuberculosis* strains*.* We found that there were at least six alleles in codon 363 of *Y. pestis aspA*. Moreover, different alleles corresponded to the activity or the inactivity of AspA [13,14]. Here, we sought to elucidate the reason why the *Y. pestis aspA* codon 363 is so polymorphous.

Initially, we proposed a hypothesis regarding the microevolution path of *Y. pestis* at the *aspA* codon 363 based on the observed polymorphisms. In this hypothesis, we applied Occam's razor theory to support the parsimony model, which explains the observations with as few evolutionary changes as possible [35,36]. Our hypothesis, grounded in Occam's razor theory, suggested an ancestral GTG allele replaced by the dominant TTG allele, alongside independent reversion mutations providing fitness advantages and forming observed polymorphisms.

To verify the above hypothesis, we compared the fitness of isogenic TTG and GTG strains by comparing their *in vitro* growth and viability under several stressed conditions. Growth capacity as an important indicator of bacterial adaptation reflects the fitness advantages of the GTG strain over the TTG strain (Figure 2(A and B)) [37,38]. Higher survival rates of the GTG strain under cold shock and anaerobic conditions also reflected stronger fitness advantages than the TTG strain (Figure 2(C and D)). Besides, the competitive fitness of bacteria can be quantified by competition assays to evaluate the adaptation of different strains in an environment with limited resources [39]. *In vitro* competition assays favoured the GTG strain, implying rapid population dominance (Figure 3). A small fraction of GTG strain cells co-cultured with TTG stain can dominate the population rapidly during successive passages, and this might account for the rapid fixations of the spontaneous mutations restoring AspA activity in the natural *Y. pestis* population. However, *in vivo* tests revealed no significant differences between TTG and GTG strain fitness (Figure 4).

We also investigated the reasons for the anomalously high proportion of the TTG stains in nature. The vast majority of bacteria can make at least one bacteriocin to create an effective barrier against competitive bacteria [40,41], and the pesticin synthesis by *Y. pestis* created an interspecies hiatus with the ancestral *Y. pseudotuberculosis* during the speciation [42]. Pesticin is the bacteriocin encoded by *pst* of *Y. pestis* pPCP_1_ plasmid that kills related bacteria of the same ecological niche, including certain strains of *Y. pseudotuberculosis* and *E*. *coli*. Notably, RNA-seq data indicated that the expression level of *pst* of the TTG strain is higher than that of the GTG strain, which suggests a higher bactericidal ability of the TTG strain. The persistence of *Y. pestis* during inter-epizootic periods remains enigmatic, while pesticin may also play a role in the post-mortem persistence by inhibiting the gut flora colonization of the extra-digestive tissues when corpse decomposition caused acidification and the temperature to ambient [43]. Furthermore, co-incubation with *Y. pseudotuberculosis* and *E. coli* cells expressing *Y. pestis* FyuA also confirmed a greater bactericidal ability of the TTG stain (Figure 5). Thus, we deduced that the higher pesticin expression level in the TTG strain conferred a competitive fitness advantage in competitive conditions. This may have partially contributed to the domination of the TTG strains in *Y. pestis* populations.

Additionally, ancient genomic data indicated that *Y. pestis* preserved the GTG as the ancestral state at *aspA*_363_ when split from *Y. pseudotuberculosis*. However, given the high proportion of the TTG strain, we speculated that a single evolutionary event may have occurred, leading to the rapid dominance of the TTG strains in the populations. The phylogenetic tree of *Y. pestis* indicated that this evolutionary event may have occurred about 4000–5000 years ago (Figure S6) [44,45], which coincided highly with the acquisition of flea-borne transmission ability (including the acquisition of *ymt* and the inactivation of *rcsA*, *ureD*, and *pde*2) [45-47]. Therefore, we hypothesized that the TTG allele occurrence and the acquisition of flea-borne transmission ability might occur within the same time frame, thus achieving rapid dominance in the populations through founder effects. However, there is no easy way to verify this assumption.

Considering all the previously mentioned findings, we have refined our evolution hypothesis, as shown in Figure 6. The GTG allele was retained at *aspA* codon 363 when *Y. pestis* speciated from *Y*. *pseudotuberculosis*. However, the TTG allele may have rapidly dominated populations through founder effects by hitchhiking on the acquisition of flea-borne transmission ability. On top of this, the TTG allele causing AspA inactivity likely provided a fitness advantage, contributing to its persistence during competition with other bacteria in nature. Nevertheless, active AspA provided fitness advantages to strains in various environments, which imposed changing selection pressures, such as cold stress and nutrient depletion, in subsequent evolution paths. As a result of the restoring AspA activity, the polymorphism at *Y. pestis aspA* codon 363 formed and exhibited its present-day appearance. Based on this hypothesis, an increasing number of new *aspA* codon 363 alleles in *Y. pestis* may emerge over time. Besides, the proportion of strains with AspA activity is significantly higher for *Y. pestis* stains isolated from vectors such as fleas (ambient temperature, more often low temperatures) than those isolated from mammals (human and rodents) (Table S7), which also implies that the proportion of AspA revertant mutant strains may continue to increase over time.

Although fitness advantages of the GTG or TTG strain have been well characterized, fitness evaluation of a GTG strain derived through mutation from a wild-type TTG strain would help solidify the causality. However, our findings demonstrate that the majority of contemporary TTG strains represent epidemic isolates. Their potential pathogenicity elevates biosafety concerns, thereby significantly complicating TTG-to-GTG mutagenesis procedures. While current experimental capacities remain unavailable, this undeniably constitutes an important direction for future research requiring immediate pursuit upon opportunity.

Although our current results support the plausibility of the hypothesis, we cannot exclude the possibility that selection for *aspA* alleles may be related to other factors, such as the ability of the bacterium to survive in soil, rodents, and fleas [48], as well as its pathogenicity towards humans or other mammals for which we do not pay much attention today. Ancient genomic data has shown that all strains of the lineages responsible for the first (0.ANT4) and second (1.PRE) plague pandemics were TTG strains. This possibly suggests that the TTG strains might be more virulent to humans than GTG strain. However, this is difficult to test due to ethical issues. Therefore, further ecological and evolutionary investigations are needed in future studies to understand the anomalous high proportion of the TTG strains with inactive AspA in natural populations and determine whether other factors or selective pressures are driving their dominance.
