# Supplementary figures and images for: Retracing the path of evolution: polymorphisms of aspA codon 363 shape the fitness of Yersinia pestis
Source: Emerg Microbes Infect. 2025 Jul 10;14(1):2532700. doi: 10.1080/22221751.2025.2532700 (PMC12291239; doi:10.1080/22221751.2025.2532700)

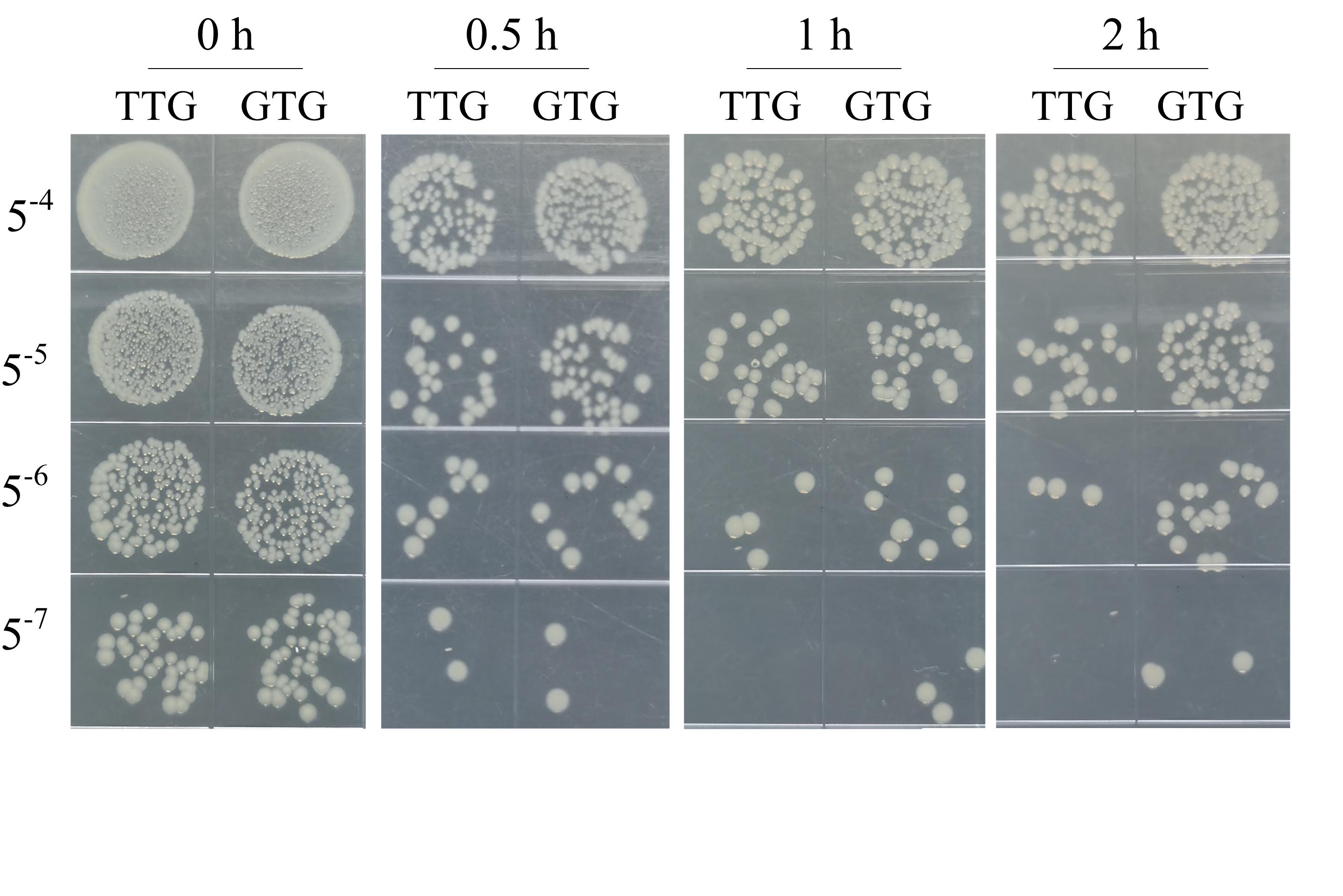

Supplement: Fig S5.jpg [file TEMI_A_2532700_SM9006.jpg]

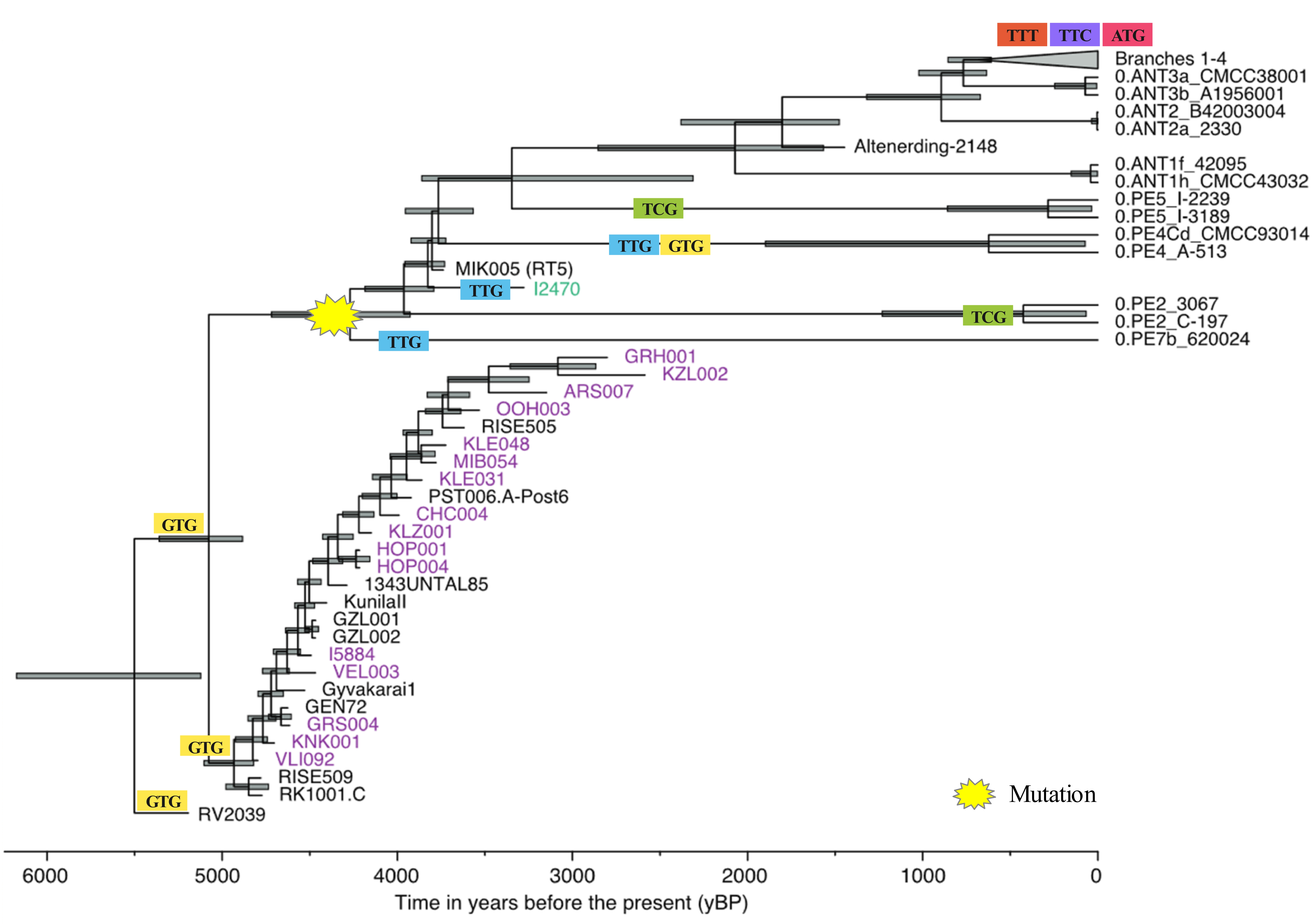

Supplement: Fig S6.jpg [file TEMI_A_2532700_SM9005.jpg]

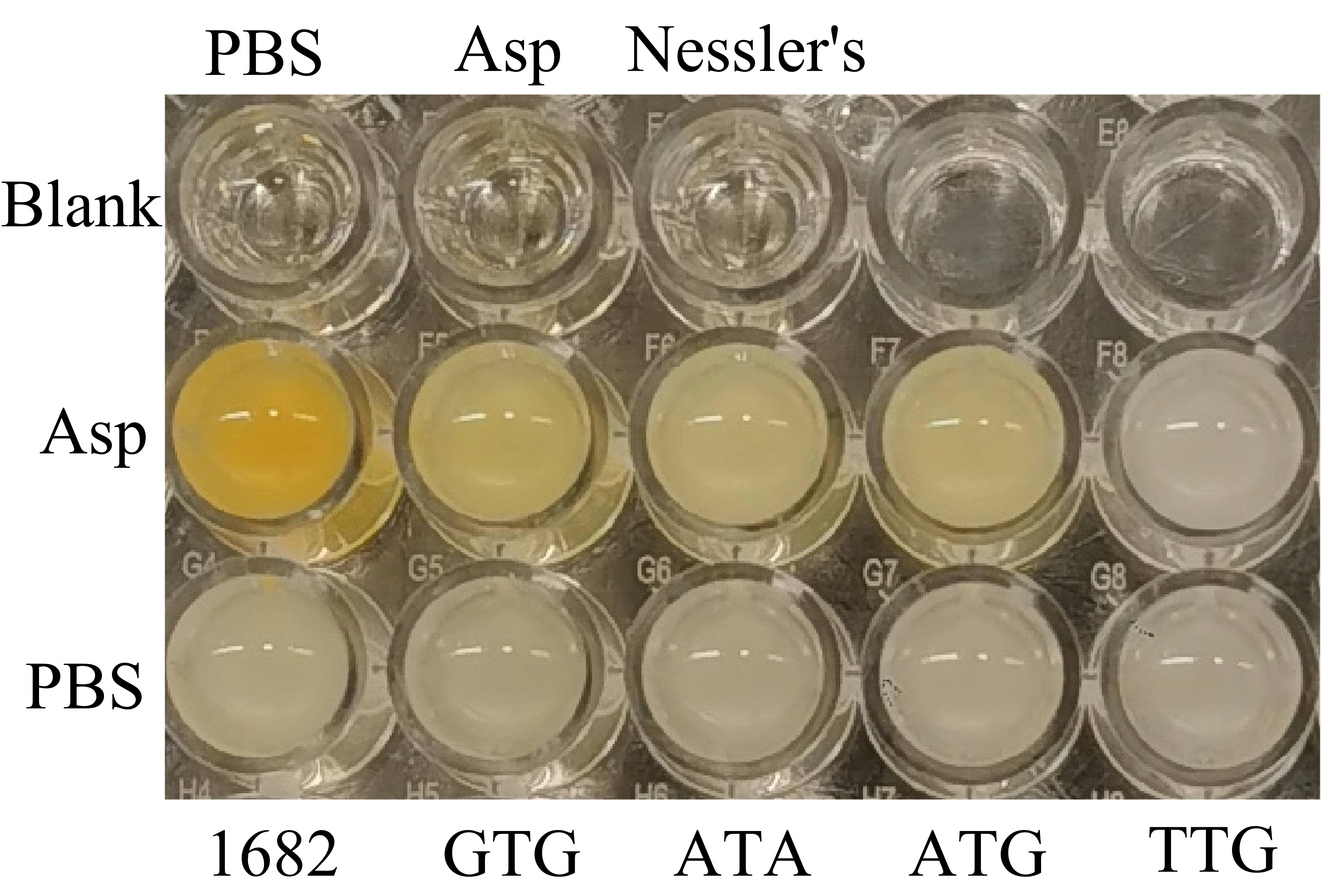

Supplement: Fig S2.jpg [file TEMI_A_2532700_SM9003.jpg]

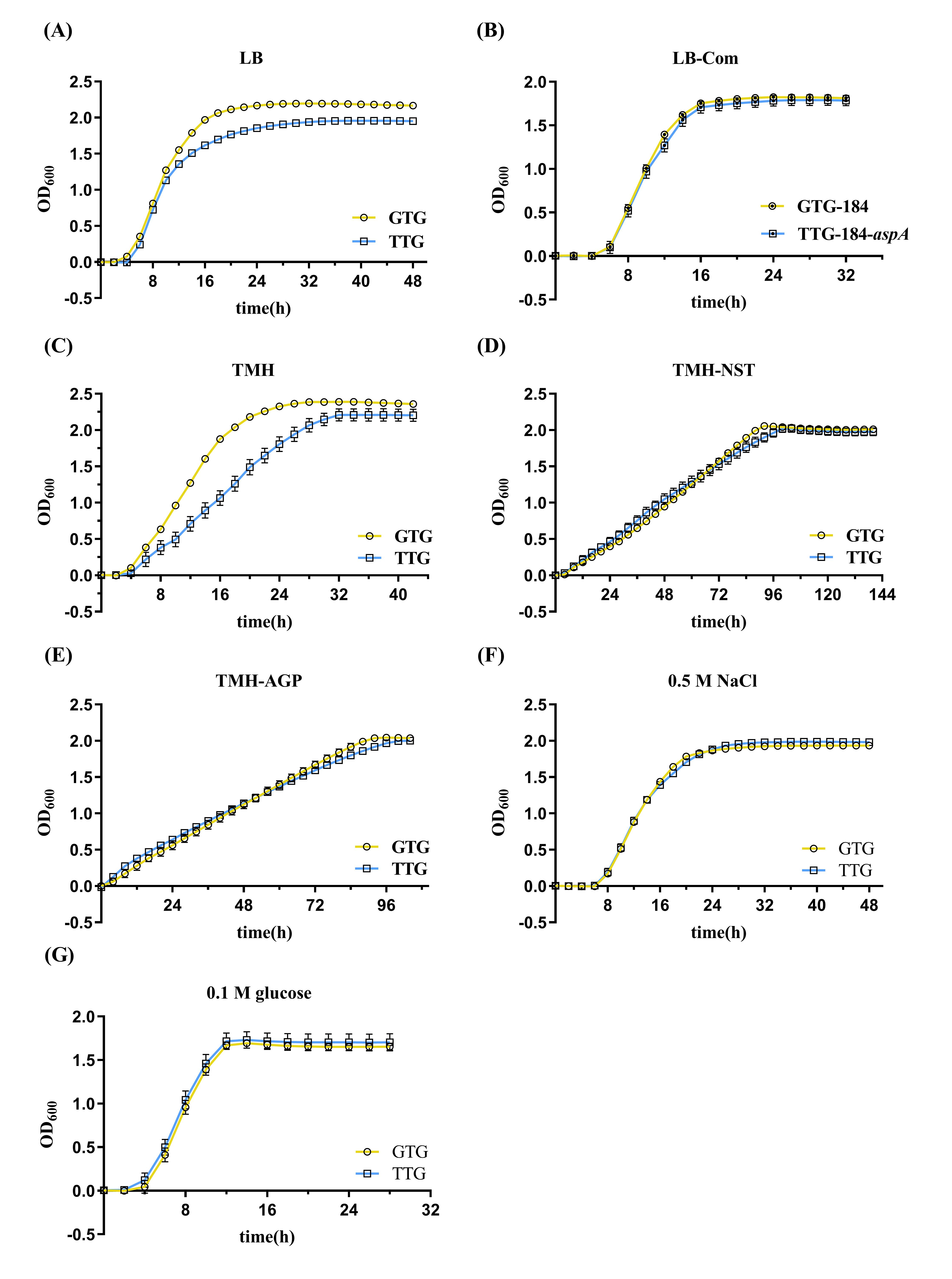

Supplement: Fig S4.jpg [file TEMI_A_2532700_SM9000.jpg]

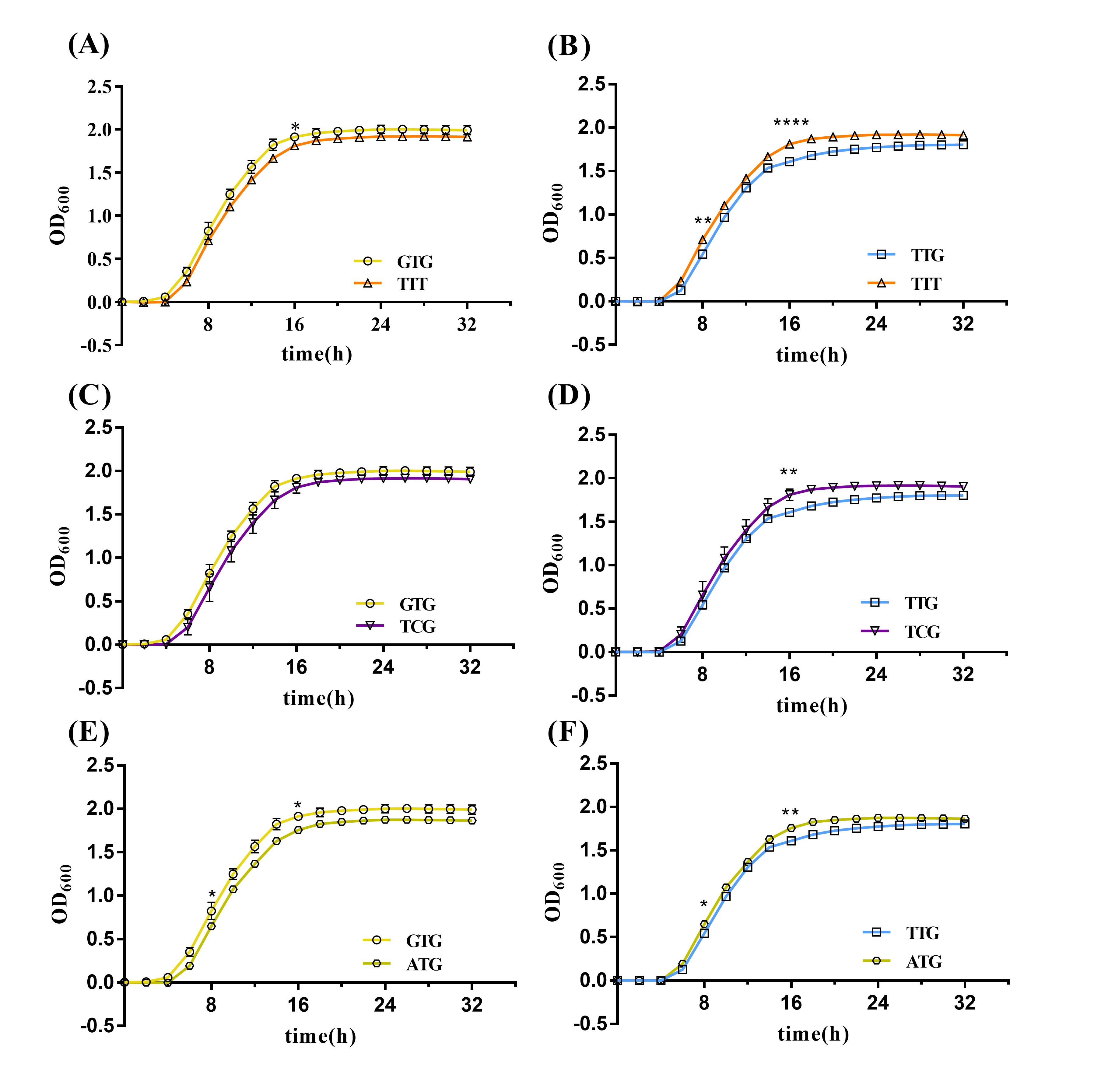

Supplement: Fig S3.jpg [file TEMI_A_2532700_SM8995.jpg]
