# Supplementary material for: Retracing the path of evolution: polymorphisms of aspA codon 363 shape the fitness of Yersinia pestis
Source: Emerg Microbes Infect. 2025 Jul 10;14(1):2532700. doi: 10.1080/22221751.2025.2532700 (PMC12291239; doi:10.1080/22221751.2025.2532700)
Supplement: Table S6.docx [file TEMI_A_2532700_SM9004.docx]

**Supplementary Table 6** Differentially expressed genes in the TTG variant compared to the GTG variant

| Gene id | Gene name | log_2_FC | p *adj* | Gene description |
| --- | --- | --- | --- | --- |
| YP_RS10800 | *virB*9 | -1.792 | 8.24E-59 | acetolactate synthase large subunit |
| YP_RS10805 | *ybtS* | -1.655 | 1.03E-14 | acetolactate synthase small subunit |
| YP_RS08755 | *virB*10 | -1.144 | 0.0011 | NADH: ubiquinone reductase (Na(+)-transporting) subunit E |
| YP_RS20425 | *nirB* | -1.107 | 9.05E-19 | NADPH-dependent FMN reductase |
| YP_RS16465 | *cspD* | -1.093 | 1.42E-15 | acetolactate synthase 2 catalytic subunit |
| YP_RS16470 | *hpaH* | -1.078 | 0.0008 | acetolactate synthase 2 small subunit |
| YP_RS00850 | *ssuE* | -1.077 | 6.02E-07 | nitrite reductase large subunit NirB |
| YP_RS16520 | *hpaI* | -1.075 | 1.10E-27 | ketol-acid reductoisomerase |
| YP_RS09420 | YP_RS12950 | -1.074 | 7.95E-24 | YadA family autotransporter adhesin |
| YP_RS00940 | *adiC* | -1.059 | 8.55E-06 | taurine ABC transporter substrate-binding protein |
| YP_RS17835 | *ilvB* | -1.038 | 8.02E-06 | copper-binding periplasmic metallochaperone CueP |
| YP_RS20420 | *ilvN* | -1.037 | 1.96E-16 | sulfonate ABC transporter substrate-binding protein |
| YP_RS10525 | YP_RS15745 | 1.003 | 2.28E-13 | hypothetical protein |
| YP_RS21535 | YP_RS21495 | 1.003 | 3.13E-06 | VirB10/TraB/TrbI family type IV secretion system protein |
| YP_RS04605 | *fliD* | 1.004 | 1.47E-06 | phage baseplate assembly protein |
| YP_RS08660 | YP_RS12205 | 1.006 | 1.59E-15 | yersiniabactin biosynthesis salicylate synthase Irp9/YbtS |
| YP_RS21525 | YP_RS05695 | 1.007 | 0.0014 | type IV secretion system protein |
| YP_RS04645 | YP_RS04600 | 1.009 | 8.08E-10 | ATP-binding protein |
| YP_RS06595 | YP_RS04630 | 1.013 | 3.38E-14 | aspartate:alanine antiporter |
| YP_RS22920 | *nqrE* | 1.016 | 9.95E-05 | zf-TFIIB domain-containing protein |
| YP_RS08500 | YP_RS10525 | 1.025 | 1.26E-06 | 5-carboxymethyl-2-hydroxymuconate Delta-isomerase |
| YP_RS12205 | YP_RS20130 | 1.031 | 1.95E-32 | YhcH/YjgK/YiaL family protein |
| YP_RS08490 | YP_RS09420 | 1.051 | 1.88E-08 | 4-hydroxy-2-oxoheptanedioate aldolase |
| YP_RS07235 | YP_RS04645 | 1.06 | 0.0001 | darobactin export ABC transporter ATP-binding protein |
| YP_RS07295 | YP_RS08500 | 1.066 | 5.58E-14 | galactoside transport ATP-binding protein |
| YP_RS04610 | *fliF* | 1.089 | 7.90E-11 | contractile injection system protein, VgrG/Pvc8 family |
| YP_RS21530 | YP_RS21490 | 1.094 | 0.0007 | P-type conjugative transfer protein VirB9 |
| YP_RS09580 | YP_RS14300 | 1.104 | 1.39E-09 | hypothetical protein |
| YP_RS06400 | YP_RS04625 | 1.113 | 8.31E-38 | cold shock-like protein CspD |
| YP_RS08495 | YP_RS09580 | 1.114 | 3.79E-12 | 2-oxo-hepta-3-ene-1,7-dioic acid hydratase |
| YP_RS07240 | YP_RS06595 | 1.123 | 2.82E-05 | darobactin export ABC transporter periplasmic adaptor subunit |
| YP_RS04640 | YP_RS04595 | 1.129 | 1.18E-10 | DUF2635 domain-containing protein |
| YP_RS04845 | YP_RS04610 | 1.129 | 7.22E-18 | putative amino acid permease |
| YP_RS08625 | YP_RS12140 | 1.15 | 9.15E-07 | yersiniabactin biosynthesis oxidoreductase YbtU |
| YP_RS04595 | *darD* | 1.154 | 2.27E-14 | baseplate J/gp47 family protein |
| YP_RS04635 | YP_RS04580 | 1.19 | 1.34E-28 | phage tail sheath subtilisin-like domain-containing protein |
| YP_RS12140 | YP_RS15750 | 1.2 | 9.06E-07 | hypothetical protein |
| YP_RS03655 | *cueP* | 1.206 | 1.53E-11 | lytic polysaccharide monooxygenase |
| YP_RS12950 | YP_RS20285 | 1.214 | 1.79E-05 | YbfA family protein |
| YP_RS15760 | YP_RS25535 | 1.219 | 0.0053 | flagellar basal-body MS-ring/collar protein FliF |
| YP_RS04600 | *darE* | 1.237 | 6.76E-06 | phage GP46 family protein |
| YP_RS05695 | *tauA* | 1.268 | 9.40E-05 | tRNA-Met |
| YP_RS20290 | *ilvM* | 1.275 | 4.26E-09 | carboxymuconolactone decarboxylase family protein |
| YP_RS07230 | YP_RS04640 | 1.282 | 0.0001 | darobactin maturation radical SAM/SPASM protein DarE |
| YP_RS22230 | YP_RS21525 | 1.29 | 3.19E-56 | pesticin |
| YP_RS04580 | *darC* | 1.29 | 2.21E-14 | phage tail protein |
| YP_RS25540 | *pst* | 1.325 | 9.75E-05 | DUF2313 domain-containing protein |
| YP_RS15750 | YP_RS22920 | 1.337 | 9.15E-06 | sigma-54 dependent transcriptional regulator |
| YP_RS04630 | *ycoA* | 1.383 | 9.61E-19 | phage tail tube protein |
| YP_RS25535 | *psaA* | 1.392 | 5.63E-06 | DUF2313 domain-containing protein |
| YP_RS04625 | *mglA* | 1.444 | 1.03E-14 | phage tail assembly protein |
| YP_RS21495 | YP_RS03655 | 1.529 | 3.00E-06 | TrbC/VirB2 family protein |
| YP_RS20285 | *ilvG* | 1.535 | 8.78E-17 | MFS transporter |
| YP_RS20130 | *ilvC* | 1.544 | 7.34E-09 | Tc toxin subunit A |
| YP_RS21490 | *ybtU* | 1.591 | 9.68E-15 | lytic transglycosylase domain-containing protein |
| YP_RS15745 | YP_RS20420 | 1.612 | 0.0005 | FliM/FliN family flagellar motor switch protein |
| YP_RS15875 | YP_RS25540 | 1.769 | 9.15E-06 | flagellar filament capping protein FliD |
| YP_RS04690 | YP_RS04605 | 2.001 | 1.67E-75 | putative outer membrane-associated protease |
| YP_RS14300 | YP_RS20290 | 2.187 | 1.02E-148 | autotransporter outer membrane beta-barrel domain-containing protein |
| YP_RS06715 | YP_RS04635 | 2.374 | 9.23E-26 | pH 6 Antigen fimbrial subunit |
