# Supplementary material for: Retracing the path of evolution: polymorphisms of aspA codon 363 shape the fitness of Yersinia pestis
Source: Emerg Microbes Infect. 2025 Jul 10;14(1):2532700. doi: 10.1080/22221751.2025.2532700 (PMC12291239; doi:10.1080/22221751.2025.2532700)
Supplement: Table S7.docx [file TEMI_A_2532700_SM9002.docx]

**Supplementary Table 7** Variation frequency of *aspA* codon 363 among strains isolated from humans, rodents, and fleas

| Source | *aspA* type | AspA activity | Number |
| --- | --- | --- | --- |
| human  or  rodent | TTG | inactive | 676 |
|  | TCG | active | 36 |
|  | TTT |  |  |
|  | GTG |  |  |
| flea | TTG | inactive | 100 |
|  | TCG | active | 12 |
|  | GTG |  |  |
|  | TTT |  |  |

Numbers of the TTG variant and other variants isolated from humans, rodents, and flea vectors among the 992 *Y. pestis* strains used to construct a phylogenetic tree. The chi-square test was used to analyze the comparison of the composition ratio between the two groups, and the *p*-value was less than 0.05.
