# Supplementary material for: Retracing the path of evolution: polymorphisms of aspA codon 363 shape the fitness of Yersinia pestis
Source: Emerg Microbes Infect. 2025 Jul 10;14(1):2532700. doi: 10.1080/22221751.2025.2532700 (PMC12291239; doi:10.1080/22221751.2025.2532700)
Supplement: Table S4.docx [file TEMI_A_2532700_SM9001.docx]

**Supplementary Table 4** Polymorphism at *aspA* codon 363 of *Y. pestis*

| **Branch** | **Phylogroup** | **TTG** | **TCG** | **GTG** | **TTT** | **ATG** | **TTC** | **Total** |
| --- | --- | --- | --- | --- | --- | --- | --- | --- |
| Branch 0 | 0.ANT1 | 82 | 3 | 0 | 1 | 0 | 0 | 86 |
|  | 0.ANT2 | 2 | 0 | 0 | 0 | 0 | 0 | 2 |
|  | 0.ANT3 | 8 | 1 | 0 | 0 | 0 | 0 | 9 |
|  | 0.ANT5 | 4 | 0 | 0 | 0 | 0 | 0 | 4 |
|  | 0.PE2 | 0 | 45 | 0 | 0 | 0 | 0 | 45 |
|  | 0.PE3 | 0 | 0 | 0 | 1 | 0 | 0 | 1 |
|  | 0.PE4 | 2 | 0 | 36 | 0 | 0 | 0 | 38 |
|  | 0.PE5 | 0 | 14 | 0 | 0 | 0 | 0 | 14 |
|  | 0.PE7 | 2 | 0 | 0 | 0 | 0 | 0 | 2 |
|  | ***0.PE8*** | 2 | 0 | 0 | 0 | 0 | 0 | 2 |
|  | ***0.ANT4*** | 14 | 0 | 0 | 0 | 0 | 0 | 14 |
|  | ***0.PRE*** | 0 | 0 | 28 | 0 | 0 | 0 | 28 |
| Branch 1 | 1.ANT | 3 | 1 | 0 | 0 | 0 | 0 | 4 |
|  | 1.IN1 | 3 | 0 | 0 | 0 | 0 | 0 | 3 |
|  | 1.IN2 | 29 | 2 | 0 | 0 | 0 | 0 | 31 |
|  | 1.IN3 | 8 | 2 | 0 | 0 | 0 | 0 | 10 |
|  | 1.IN5 | 19 | 0 | 0 | 0 | 0 | 0 | 19 |
|  | 1.ORI1 | 47 | 1 | 0 | 1 | 0 | 0 | 49 |
|  | 1.ORI2 | 474 | 0 | 0 | 2 | 1 | 0 | 477 |
|  | 1.ORI3 | 51 | 0 | 0 | 0 | 0 | 0 | 51 |
|  | ***1.PRE*** | 49 | 0 | 0 | 0 | 0 | 0 | 49 |
| Branch 2 | 2.ANT1 | 4 | 0 | 0 | 1 | 0 | 0 | 5 |
|  | 2.ANT2 | 6 | 0 | 0 | 0 | 0 | 0 | 6 |
|  | 2.ANT3 | 14 | 3 | 0 | 1 | 0 | 0 | 18 |
|  | 2.MED0 | 6 | 0 | 0 | 1 | 0 | 0 | 7 |
|  | 2.MED1 | 57 | 0 | 0 | 0 | 0 | 0 | 57 |
|  | 2.MED2 | 5 | 0 | 0 | 0 | 0 | 1 | 6 |
|  | 2.MED3 | 24 | 0 | 1 | 0 | 0 | 0 | 25 |
| Branch 3 | 3.ANT1 | 1 | 3 | 0 | 0 | 0 | 0 | 4 |
|  | 3.ANT2 | 5 | 3 | 0 | 0 | 0 | 0 | 8 |
| Branch 4 | 4.ANT1 | 10 | 0 | 0 | 1 | 0 | 0 | 11 |
| Total | -- | 931 | 78 | 65 | 9 | 1 | 1 | 1085 |

Polymorphism at *aspA* codon 363 of 1085 published *Y. pestis* strains. The ancient DNA samples are marked in bold and italic.
