# Supplementary material for: Retracing the path of evolution: polymorphisms of aspA codon 363 shape the fitness of Yersinia pestis
Source: Emerg Microbes Infect. 2025 Jul 10;14(1):2532700. doi: 10.1080/22221751.2025.2532700 (PMC12291239; doi:10.1080/22221751.2025.2532700)
Supplement: Table S1.docx [file TEMI_A_2532700_SM8998.docx]

**Supplementary Table 1** Bacterial strains and plasmids used in this study

| **Strain or plasmid** | **Features** | **Source** |
| --- | --- | --- |
| ***E. coli*** |  |  |
| S17-λ*pir* | *recA,* *λpir* | ^1,2^ |
| S17-pDS132-Mut | pDS132-Mut was introduced into S17-λpir | This study |
| DH5α-*fyuA* | DH5α-*fyuA* was introduced into DH5α | This study |
| ***Y. pestis*** |  |  |
| 201-GTG | biovar Microtus strain 201, wild type | ^3^ |
| 201-TTG | a TTG mutation was introduced in 201-GTG at the at *aspA* codon_363_ | This study |
| 201-TCG | a TCG mutation was introduced in 201-GTG at the at *aspA* codon_363_ | This study |
| 201-TTT | a TTT mutation was introduced in 201-GTG at the at *aspA* codon_363_ | This study |
| 201-ATG | an ATG mutation was introduced in 201-GTG at the at *aspA* codon_363_ | This study |
| GTG-184 | pACYC184 was introduced into 201-GTG | This study |
| TTG-184-*aspA* | pACYC184-*aspA* was introduced into 201-TTG | This study |
| ***Y. pseudotuberculosis*** |  |  |
| 1 a | *Y. pseudotuberculosis* strain of O: 1a type | This study |
| 1 b | *Y. pseudotuberculosis* strain of O: 1b type | This study |
| **plasmids** |  |  |
| pDS132 | Suicide vector, *cat* | ^4^ |
| pACYC184 | Cloning vector, Cm^r^ Tet^r^ | ^5^ |
| pDS132-Mut | *aspA* fragments with mutation at codon_363_ were inserted into pDS132 | This study |
| pACYC184-*aspA* | *aspA* was inserted into pACYC184, used for the complement of *aspA* | This study |
| DH5α-*fyuA* | *fyuA* was introduced into DH5α | This study |

1 Brem, D., Pelludat, C., Rakin, A., Jacobi, C. A. & Heesemann, J. Functional analysis of yersiniabactin transport genes of *Yersinia enterocolitica*. *Microbiology* **147**, 1115-1127, doi:10.1099/00221287-147-5-1115 (2001).

2 Simon, R., Priefer, U. & Pühler, A. Genetic Engineering: Transposon Mutagenesis in Gram Negative Bacteria. *Bio/Technology* (1983).

3 Song, Y. *et al.* Complete genome sequence of Yersinia pestis strain 91001, an isolate avirulent to humans. *DNA Res* **11**, 179-197, doi:10.1093/dnares/11.3.179 (2004).

4 Philippe N, A. J. P., Coursange E, Geiselmann J, Schneider D. Improvement of pCVD442, a suicide plasmid for gene allele exchange in bacteria. *Plasmid* **51**, 246-255, doi:10.1016/j.plasmid.2004.02.003 (2004).

5 Chang, A. Construction and characterization of amplifiable multicopy DNA cloning vehicles derived from the P15A cryptic miniplasmid. *Journal of Bacteriology* **134** (1978).
