# Supplementary material for: Retracing the path of evolution: polymorphisms of aspA codon 363 shape the fitness of Yersinia pestis
Source: Emerg Microbes Infect. 2025 Jul 10;14(1):2532700. doi: 10.1080/22221751.2025.2532700 (PMC12291239; doi:10.1080/22221751.2025.2532700)
Supplement: Table S2.docx [file TEMI_A_2532700_SM8997.docx]

**Supplementary Table 2** Defined TMH medium and the TMH with removal of different amino acids^#^

|  | Ingredients (MW) | Storage fluid (mM) | Concentration (mM) |
| --- | --- | --- | --- |
| Salt solution | K_2_HPO_4_·3H_2_O (228.22) | 25 | 2.5 |
|  | [citric acid](javascript:;) (192.14) | 100 | 10.0 |
|  | NH_4_Cl (53.49) | 100 | 10.0 |
|  | MgCl_2_·6 H_2_O (203.30) | 200 | 20 |
|  | MnCl_2_·4 H_2_O (197.92) | 0.1 | 0.01 |
| Stable amino acid solution | DL-Alanine (89.09) | 5 | 2.5 |
|  | L-Leucine (131.2) | 2 | 1.0 |
|  | L-Tyrosine (181.19) | 2 | 1.0 |
|  | L- Isoleucine (131.2) | 2 | 1.0 |
|  | L-Valine (117.15) | 2 | 1.0 |
|  | L-Phenylalanine (165) | 2 | 1.0 |
|  | L-Threonine (119.13) | 5 | 2.5 |
|  | L-Histidine (155.16) | 2 | 1.0 |
|  | L-Arginine (174) | 2 | 1.0 |
|  | L-Aspartate (133.11) | 2 | 1.0 |
|  | L-Methionine (149.21) | 2 | 1.0 |
|  | L-Proline (115.14) | 10 | 5.0 |
|  | L-Lysine (146.19) | 2 | 1.0 |
|  | L-Glutamic acid (147.13) | 10 | 5.0 |
|  | Glycine (75.07) | 10 | 5.0 |
|  | L-Serine (105.09) | 10 | 5.0 |
| Unstable amino acid solution | L-Tryptophan (204.23) | 10 | 0.1 |
|  | L-Asparagine (150.13) | 125 | 2.5 |
|  | L-Glutarnine (146.15) | 50 | 1.0 |
| Vitamin solution | V_B1_ (337.3) | 30 | 0.003 |
|  | V_B5_ (238.3) | 40 | 0.004 |
|  | V_H_ (244.3) | 20 | 0.002 |
| Others (g/L) | Na_2_S_2_SO_3_·5 H_2_O (248.17) | 0.620425 g | 2.5 |
|  | HEPES (239.312) | 5.9575 g | 25 |
|  | [potassium gluconate](javascript:;) (234.25) | 2.342 g | 10 |
|  | CaCl_2_ (110.984) | 0.27748 g | 2.5 |
|  | FeSO_4_·7H_2_O (278.01) | 0.027802 g | 0.1 |

Adjust the pH to 7.2.

#: TMH-NST, TMH without NH_4_^+^, L-Proline, L-Serine, L-Threonine, L-Glutamate, and L-Aspartate. TMH-AGP, TMH without L-Aspartate, L-Asparagine, L-Glutamate, L-Glutarnine, and L-Proline.
